# Optimal Solution to the Torsional Coefficient Fitting Problem in Force Field Parametrization

**DOI:** 10.1021/acs.jpca.0c10845

**Published:** 2021-03-24

**Authors:** Adrian Kania, Krzysztof Sarapata, Michał Gucwa, Anna Wójcik-Augustyn

**Affiliations:** Department of Computational Biophysics and Bioinformatics, Faculty of Biochemistry, Biophysics and Biotechnology, Jagiellonian University, ul. Gronostajowa 7, 30-387 Cracow, Poland

## Abstract

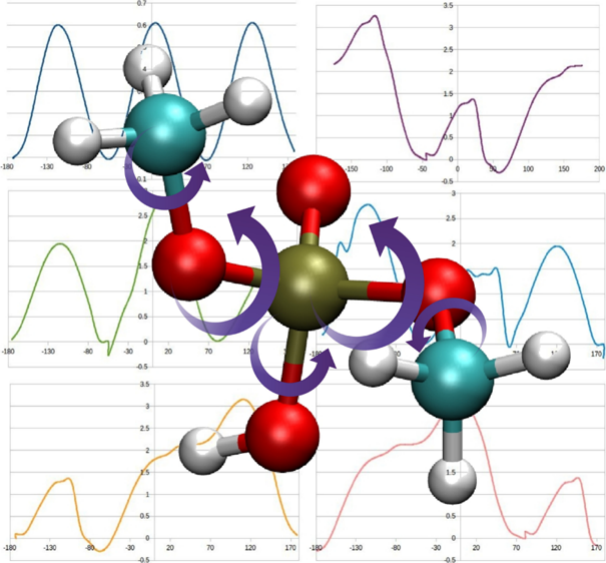

Molecular modeling is an excellent tool for studying biological systems on the atomic scale. Depending on objects, which may be proteins, nucleic acids, or lipids, different force fields are recommended. The phospholipid bilayers constitute an example, in which behavior is extensively studied using molecular dynamics simulations due to limitations of experimental methods. The reliability of the results is strongly dependent on an appropriate description of these compounds. There are some deficiencies in the parametrization of intra- and intermolecular interactions that result in incorrect reproduction of phospholipid bilayer properties known from experimental studies, such as temperatures of phase transitions. Refinement of the force field parameters of nonbonded interactions present in the studied system is required to close these discrepancies. Such parameters as partial charges and torsional potential coefficients are crucial in this issue and not obtainable from experimental studies. This work presents a new fitting procedure for torsional coefficients that employs linear algebra theory and compares it with the Monte Carlo method. The proposed algebraic approach can be applied to any considered molecular system. In the manuscript, it is presented on the example of dimethyl phosphoric acid molecule. The advantages of our method encompass finding an optimal solution, the lack of additional parameters required by the algorithm, and significantly shorter computational time. Additionally, we indicate the importance of proper assignment of the partial charges.

## 1 Introduction

Molecular modeling is an indispensable part of the research performed on innovative materials and biological systems, such as proteins, nucleic acids, or phospholipid bilayers. The molecular dynamics (MD) simulations are a valuable tool to analyze the interactions within the studied system, investigate its thermodynamic properties, and follow the diffusion process or conformational transitions. Importantly, MD belongs to the semi-empirical force field (FF) methods, which provide the description of the system framed based on the available experimental and theoretical studies. Therefore, such an approach makes the obtained results strongly dependent on the quality of the applied FF, which yield many limitations into the potential range of applications. FFs such as AMBER and CHARMM are developed for studying proteins and nucleic acids,^1−4^ while optimized potentials for liquid simulation force field, all-atom (OPLS-AA), and GAFF are generic FFs dedicated to small organic molecules, containing a wide range of functional groups.^5−7^

**Figure 1:**
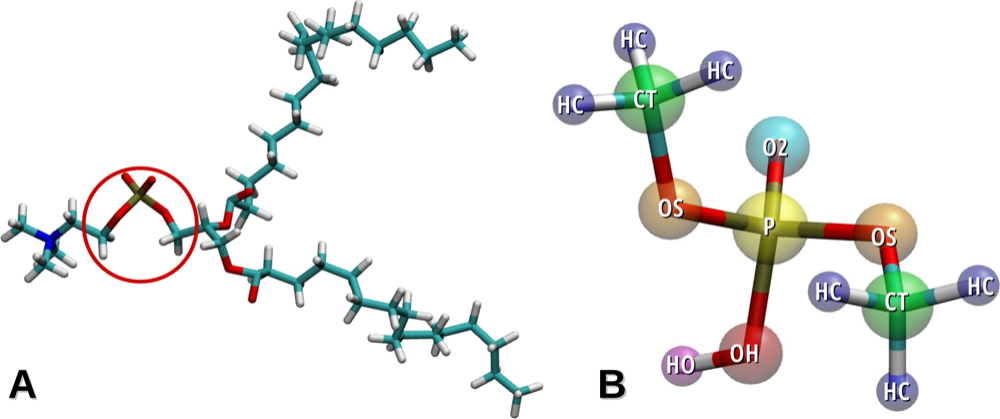
(A) Structure of the phosphatidylcholine molecule with marked chemical groups present in the dimethyl phosphoric acid; (B) optimized structure of dimethyl phosphoric acid. Different colors represent different atom types as follows: in blue HC, in green CT, in orange OS, in yellow P, in cyan O2, in red OH and in pink HO.

A FF is a set of parameters assigned to specific atom types and formulas describing interatomic interactions, which are applied in the MD simulations. FF encompasses the formula for potential energy and its parameters (eq 1). It is a semi-theoretical approach as it demands a set of constants, which may be derived from experimental studies or quantum mechanical (QM) calculations.^8,9^ Using FF, we assume additivity and transferability. The first term means that the complete energy of a system may be considered as a sum of individual potentials representing bonded and nonbonded interactions. Transferability means that the FF, which was developed for small molecules, can be applied to larger complexes that contain the same model components.^1^ For example, when considering parameters for phospholipid molecules, we may assign them to the small model molecules that include the corresponding moieties, that is, dimethyl phosphoric acid contains chemical groups present in the phospholipid polar head (Figure 1A).

In the complex systems, such as phospholipid bilayers, where the intermolecular interactions determine the properties of the system, proper assignment of parameters determining nonbonded interactions seems to be significant. Many FF revealed insufficient description of nonbonded interactions and thus discrepancies in the reproduction of experimentally known properties (membrane thickness, deuterium order parameters, or temperature of phase transition) by models of phospholipid bilayers.^1,10−12^ The estimation of nonbonded parameters poses a lot of problems. Nonbonded interactions resulting from partial charges and torsional parameters assignment are not possible to estimate in experimental studies; thus, QM methods are usually applied. At this point, the problem of torsional parameters fitting to the QM energy profiles for rotation of dihedral angles appears. The problem results from the fact that the coefficients of the torsional potential of each dihedral angle present in the molecule affect to a greater or lesser degree the torsional potential of other dihedral angles. More specifically, the good reproduction of the QM energy profile for one dihedral angle can cause deterioration of reproduction of energy profiles for other dihedral angles. Therefore, the best solution is to proceed the fitting procedure for torsional potentials of all dihedral angles presented in the considered molecule, simultaneously. There are many approaches for this purpose, including genetic algorithms, the simplex, Monte Carlo methods, and many others.^8,13−16^ We propose an algebraic solution to this problem. We indicate a global solution and offer some modification for obtaining parameters in a specific range. Moreover, we show that proper assignment of atomic partial charges significantly improves the quality of fitting.

In this paper, we present our algebraic method of torsional parameters fitting on the example of the dimethyl phosphoric acid molecule (DMPH), which constitutes a model molecule for phospholipid parametrization, as well as an important component of the innovative ionic liquids used in petrochemistry.^17^ The assigned parameters were tested via MD simulations and validated for the ability to reproduce the condensed phase properties known from experimental studies.

## 2 Models and Methods

### 2.1 DMPH Molecule

In the present study, dimethyl phosphoric acid (DMPH) is used as a model molecule. DMPH contains chemical moieties (atom types) present in the hydrophilic part of phospholipids (Figure 1A). Moreover, ionic liquids containing dimethyl phosphoric acid is considered as highly efficient in petrochemistry because of their high dissolution capacity.^17^ DMPH is a small, water-soluble organic molecule that belongs to the class of dialkyl phosphates. There are two protonation states available for dimethyl phosphate molecule, in which phosphate oxygen is protonated (DMPH) or not (DMP^–^). For DMPH, the experimentally measured p*K*_a_ of 1.29^18^ indicates that it mainly occurs in its anionic form. However, it is worth to mention that the experimental studies^18^ revealed dependence between p*K*_a_ and length of alkyl substituents in dialkyl phosphates, which increases the probability of appearance of the protonated form of the phosphate moiety in phospholipids or ionic liquids. We tested our procedure on the protonated form of dimethyl phosphoric acid, because for such a state, according to FF parametrization philosophy,^19^ we are able to check the influence of the refined parameters on the condensed phase properties of DMPH—known from experimental studies, such as density and enthalpy of vaporization. Reproduction of these properties strongly depends on the proper description of intermolecular interactions.

The DMPH molecule consists of 14 atoms, which can be grouped into following seven atom types: HC, CT, OS, P, O2, OH, and HO (Figure 1B). Within the DMPH molecule, there are six types of dihedral angles, which are described by the atom types that compose them (Table.1).

**Table 1:** Dihedral Types within the DMPH Molecule

dihedral type	atom type	atom type	atom type	atom type
1	CT	OS	P	OS
2	CT	OS	P	O2
3	CT	OS	P	OH
4	HC	CT	OS	P
5	HO	OH	P	O2
6	HO	OH	P	OS

### 2.2 FF Formula

A FF describes the potential energy of the system. It includes both expressions describing individual contributions of each component to the total energy as well as relevant parameters. There are two types of interactions: bonded and nonbonded, which constitute the total energy of the system (eq 1). The nonbonded interactions are provided by two terms, that is, Coulomb and Lennard-Jones potentials (eq 3), while bonded interactions are described by three terms, that is, harmonic potentials for bond stretching and bending of angles and torsional potential for conformational transitions of dihedral angles (eq 2).
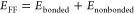
1
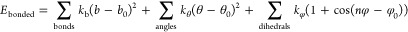
2
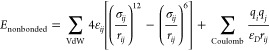
3

In the term describing the energy of conformational changes of the dihedral angle in eq 2, *k*_φ_ determines the coefficients of Fourier series. This equation can be rewritten according to the representation of torsional potential used by most of the packages applied in MD simulations into eq 4.
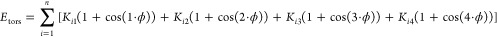
4where . In the OPLS-AA FF, also Ryckaert-Bellemans function might be used to describe torsional potential (eq 5).
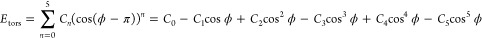
5

In the present study, we propose the algorithm assigning the Fourier coefficients. However, they can be easily converted to the coefficients of Ryckaert-Bellemans function.

### 2.3 Fourier to Ryckaert-Bellemans Transformation

Below, we present a transformation of torsional potential from Fourier series (eq 4) to Ryckaert-Bellemans function (eq 5). In the OPLS-AA FF function of dihedral potential is given by four terms of Fourier series as in eq 6, which might be easily transformed through eqs 7 and 8 to the final form of the function represented by eq 9.
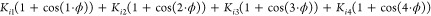
6
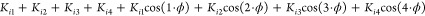
7
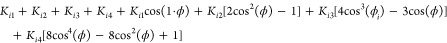
8
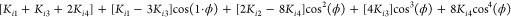
9

Comparing eq 9 with the Ryckaert-Bellemans potential (eq 5) allows us to determine Ryckaert-Bellemans coefficients as functions of the Fourier coefficients (eqs 10–15).
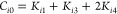
10
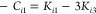
11
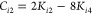
12
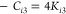
13
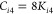
14
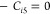
15

### 2.4 Parametrization of the Torsional Potential

Potential energy can be expressed as the sum of potential energy without the torsional energy component (*E*_zero–tors_) and torsional energy part (*E*_tors_), as is written in eq 16.
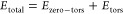
16
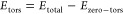
17

The energy of a system deprived of components from torsional potential (*E*_zero–tors_) might be estimated using the *grompp* program from the Gromacs package^20^ by setting all torsional parameters to zero. The reference potential energy *E*_tot_ can be computed using QM methods. Therefore, torsional energy potential is estimated as a function constituting the difference between the profiles for the rotation of dihedral angles obtained with QM methods and corresponding profiles of *E*_zero–tors_ obtained with the Gromacs package (or other used). The formula of the function describing the dihedral energy term is known (eq 4); thus, the parameters might be estimated by the minimization process (eq 18).
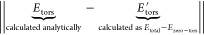
18

In the present study, for each dihedral angle, 36 conformers of the model molecule were optimized with QM methods. Each conformer is characterized by the selected dihedral angle remaining in the range from −180 to 180°, with a step of 10°. For example, for *n* dihedrals considered, 36*n* torsional energies are applied. In the case of DMPH molecule, we consider 15 dihedral angles, and thus, 540 conformers and their torsional energies were used. It is worth to mention here that for each of these 540 geometries, all 15 dihedral angles are measured, and thus for each dihedral angle, all 540 values must be considered in the torsional coefficients fitting procedure. Realizing that one geometry of the DMPH molecule constituting a set of 15 dihedral angles values is important to understand that profiles for all dihedral angles are conjugated, and thus, all torsional coefficients characterizing a small-model molecule should be assigned simultaneously. The scan of each dihedral angle present in the molecule is required to provide a wide range of configurations and to avoid overfitting problem.^21^ Taking into account that each dihedral type is described by 4 coefficients and 15 dihedrals within the DMPH molecule are grouped into 6 dihedral types (Table 1), 24 parameters are required to describe torsional potential for the DMPH molecule. These torsional coefficients were obtained using the minimization procedure (eq 19), where *F*(*K*) represents difference between assigned torsional potential and reference derived from QM calculations (eq 20).
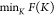
19
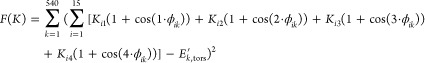
20

### 2.5 Monte Carlo Method

**Figure 2:**
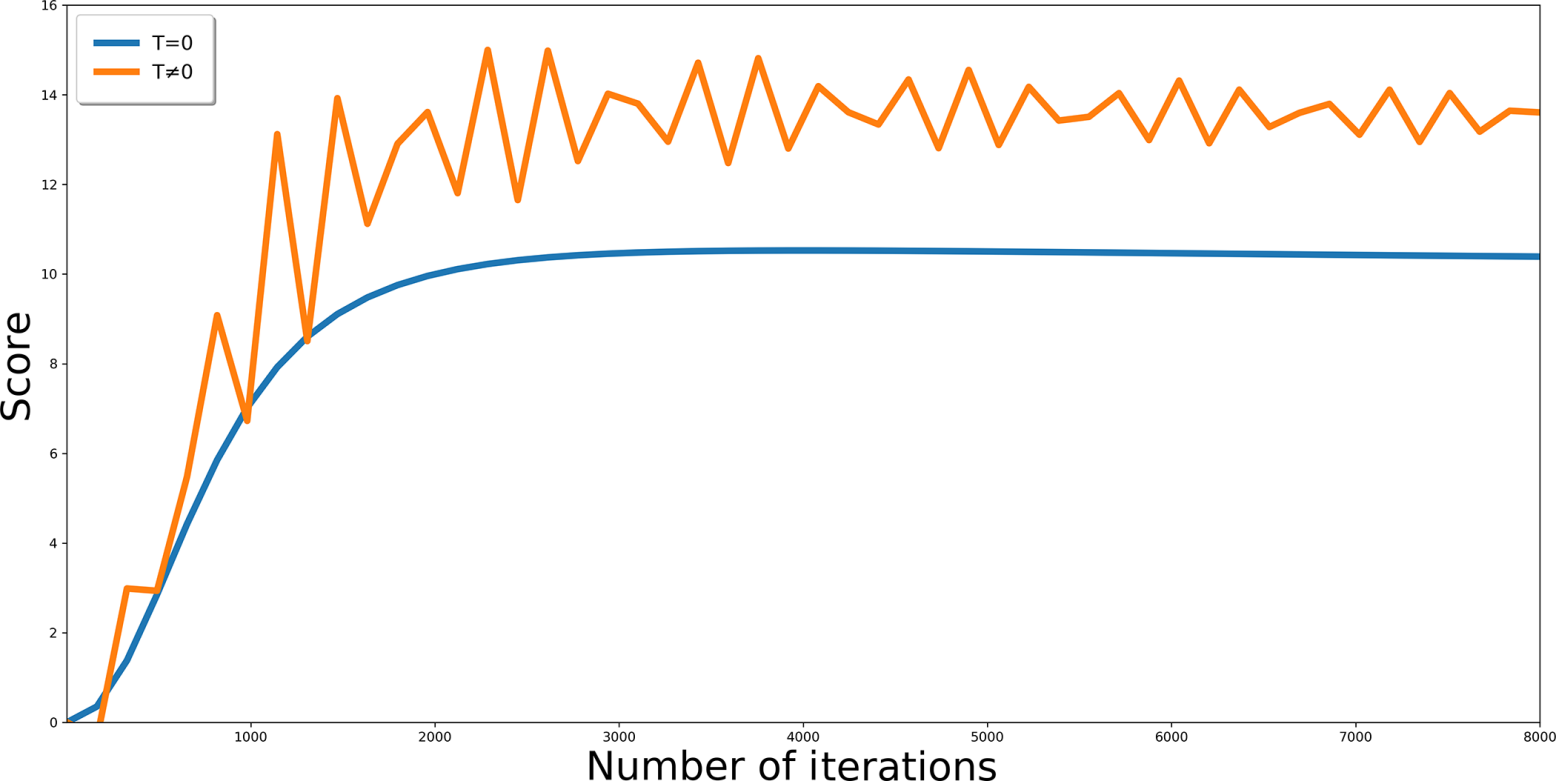
Influence of temperature factor on the convergence of the Monte Carlo minimization algorithm. Using *T* parameter enables obtaining better score and, as a result, a more optimal solution.

The general scheme of the Monte Carlo methods is based on the following idea. To find a minimum of a function *F*, the probability transition from a state *F*_*n*_ to *F*_*n*+1_ is determined by *P*_*T*_ given in eq 22, where the *T*-factor prevents too early convergence and reaching the local minimum (Figure 2).
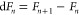
21
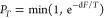
22

According to eq 22, if *F*_*n*+1_ < *F*_*n*_, then *P*_*T*_ = 1, and in the subsequent step, the value is set to *F*_*n*+1_. On the other hand, if *F*_*n*+1_ > *F*_*n*_, d*F* > 0, then *P*_*T*_ = *e*^–d*F*_n_/*T*^∈(0,1) (the probability is greater than zero). The *K*_*ij*_ values in eq 20, which determine that *F*_*n*_ are usually generated using random-number generators and iteratively modified. Score function is computed for each iteration (Figure 2).

### 2.6 Analytical Solution

In our analytical solution, we reconsider eq 20, which might be subsequently rewritten to eq 23, where *A*_*ikj*_ = 1 + cos(*j*Φ_*ik*_) are known.
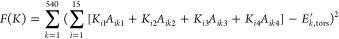
23

Some of the dihedral angles are assigned to the same group type, collected in [2, 2, 2, 6, 1, 2]. The first two angles are from the same group; so, *K*_11_ = *K*_21_ = *k*_1_^1^ (first coefficient for the first dihedral type); next two angles are from another group; so, *K*_12_ = *K*_22_ = *k*_2_^1^ (second coefficient for the first dihedral type); and so forth (a total of six groups separated by commas). As a consequence, eq 24 might be written.
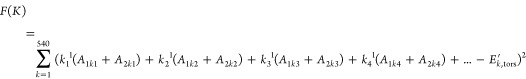
24

The function *F* is differentiable, non-negative, and thus has a global minimum, as it is a sum of squares of linear expressions. Searching for extremum, we write necessary conditions: δ*F*/δ*k*_*i*_^j^ = 0. For example
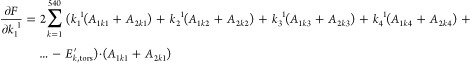
25the condition
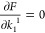
26implicates
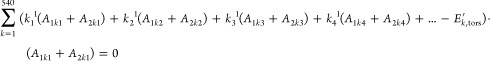
27which can be rewritten as follows
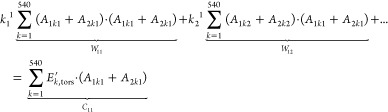
28and shortly as follows
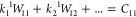
29

Similar equations may be written for 23 remaining conditions (namely, ) and, consequently, this system of linear equations may be shortly presented as *WK* = *C*, where , , , where *W*_*ij*_ and *C*_*ij*_ depend only on *A*_*ikj*_ and *E*_*k*,tors_. This system can be resolved analytically, and optimal torsion parameters *k*_*i*_j may be obtained during so-called “global” minimization (e.g., *K* = *W*^–^^1^*C*, if *W* is reversible). If a certain range for *k*_*i*_^j^ parameters is required, the problem can be formulated with eq 30, where *T* is a given real number (in the present study, we tested this approach for *T* = 20 and called it “local minimization”). Both procedures, that is, global and local minimization, with all details, are coded in a Python script called *K_fit.py* provided as Supporting Information to this manuscript. They may be applied to other molecule in future studies.
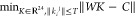
30

### 2.7 QM Calculations: Energy Profiles of Dihedral Angles and Procedure of Point Charges Fitting

QM calculations for protonated DMPH were performed with Gaussian 09.^22^ All geometries were optimized using the density functional theory method combining the hybrid exchange–correlation B3LYP functional with D3 Grimme’s dispersion correction^22−24^ and cc-pVTZ triple-ζ basis set. The energy scans were performed for all dihedral angles present in the DMPH molecule (15), and during geometry optimization, the constraints were imposed only on the scanned dihedral angle. In the DMPH, there are 15 dihedral angles, which might be assigned to 6 types of dihedral angles, as follows: 2 dihedral angles are characterized by the CT-OS-P-OS type, 2 dihedrals of the CT-OS-P-OH type, 2 dihedrals of the CT-OS-P-O2 type, 6 dihedrals of the HC-CT-OS-P type, 1 dihedral of the O2-P-OH-HO type, and 2 dihedrals of the OS-P-OH-HO type (Table 1). For each of the fifteen dihedral angles, the energy scan was performed with a step of 10° to obtain 36 conformers characterized by values of each angle in the range from −180 to 180°. This procedure provided 540 conformers of protonated dimethyl phosphoric acid, in which energies and geometries were applied to the presented procedure developed to fit dihedral Fourier coefficients.

For the most stable geometry of the DMPH molecule, point charges were assigned according to the RESP procedure based on the electrostatic potential computed on the B3LYP-D3/cc-pVTZ level with Gaussian 09.^22,25^ In order to maintain the transferability of parameters, additional nonbonding atom types were not introduced, and thus, partial charges were assigned to the existing FF atom types.

### 2.8 MM Calculations: Energy Profiles of Dihedral Angles and Parameter Validation

Classical MD simulations for gas and condensed phases of dimethyl phosphoric acid were performed with Gromacs v 5.1.2^20^ using OPLS-AA FF.^26^ We tested several sets of parameters that differed in van der Waals parameters, partial charges, and dihedral coefficients. More specifically, two sets of van der Waals parameters were tested: ORG—known from OPLS-AA FF^26^ and M set—provided by Murzyn et al. for the DMPH molecule,^19^ three sets of partial charges were tested, that is, M set—proposed by Murzyn et al.,^19^ Qopls set—original OPLS-AA charges, Q0 set—obtained from QM calculations, and RESP procedure. Dihedral parameters were fitted using *fit_dihedral.py* program^14^ and algebraic approach for each set combining partial charges and van der Waals parameters (Table 2). All energy profiles for rotation of 15 dihedral angles, that is, for parameter sets with all torsional coefficients set to zero and for all sets with assigned new torsional coefficients, were calculated for optimized geometries derived from QM calculations (single-point calculations).

**Table 2:** Considered Sets of Parameters

set name	set type	vdW parameters	charge values	minimization type	number of parameters
set 1	1	ORG	Q0	L	3
set 2	1	ORG	Q0	G	3
set 3	1	ORG	Q0	L	4
set 4	1	ORG	Q0	G	4
set 5	2	ORG	Qopls	L	4
set 6	2	ORG	Qopls	L	3
set 7	2	ORG	Qopls	G	4
set 8	2	ORG	Qopls	G	3
set 9	3	M	Q0	L	3
set 10	3	M	Q0	G	3
set 11	3	M	Q0	L	4
set 12	3	M	Q0	G	4
set 13	4	M	M	L	3
set 14	4	M	M	G	3
set 15	4	M	M	L	4
set 16	4	M	M	G	4

For two sets of parameters labeled in Table 2 as set 4 and set 12, which gave the best results of fitting, the molecular mechanic (MM) geometry optimization using the steepest descent method with restraints imposed on the particular dihedral angle (force constant of 10^8^ kJ/mol/rad^2^) was performed for 540 geometries of DMPH obtained from QM calculations.

The main purpose of refinement of the DMPH parameterization was improving the description of interatomic and intermolecular interactions and thus better reproduction of physicochemical properties by MD simulations. We considered two condensed phase properties: density and enthalpy of vaporization. The second one was calculated according to eq 31, where *R* is the gas constant and *N* is the number of molecules in the simulation box (*N* = 800).
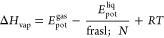
31

In the gas-phase MD simulations, a stochastic dynamics algorithm was used for integrating the equations of motion. The temperature of simulations lasting 1000 ns was set to 298 K. We put the inverse friction constant 1 ps during temperature coupling. We calculated the average value of potential energy from the equilibrated part of trajectory 150–1000 ns.

The MD simulations for the condensed phase of DMPH were performed in the periodic boundary conditions. Newtonian leapfrog integrator was applied. We employed the Nosé–Hoover thermostat for temperature coupling. The temperature of simulations was set to 298 K. The temperature and pressure coupling constants were set to 0.5 and 5.0 ps. Particle-mesh Ewald summation was used for electrostatic interaction description (with cut off = 1 nm). The simulation box consisted of 800 DMPH molecules. The box had an average length of 5 nm. The MD simulations were performed in two steps: first 20 ns in canonical ensemble (*NVT*), subsequent 50 ns in the isothermal-isobaric (*NPT*) ensemble, and finally, for additional 150 ns of *NPT* simulations, we calculated the average value of potential energy and density using the *gmx energy* program from the Gromacs package.^20^

## 3 Results and Discussion

We fitted dihedral parameters for four different sets of partial charges and van der Waals parameters. All sets are gathered in Table 2. We applied both “global” and “local” (the values of Fourier coefficients are limited to the range from −20 to 20) minimization procedures. For each set of partial charges and van der Waals parameters, we assigned three or four coefficients for one dihedral type. We considered the original OPLS-AA charges (*Q*_opls_), the modification of them proposed by Murzyn et al. (M),^19^ and those obtained from the RESP procedure and QM calculations (Q0). Two sets of van der Waals parameters were tested: the original values and those proposed by Murzyn et al. (M).^19^

**Figure 3:**
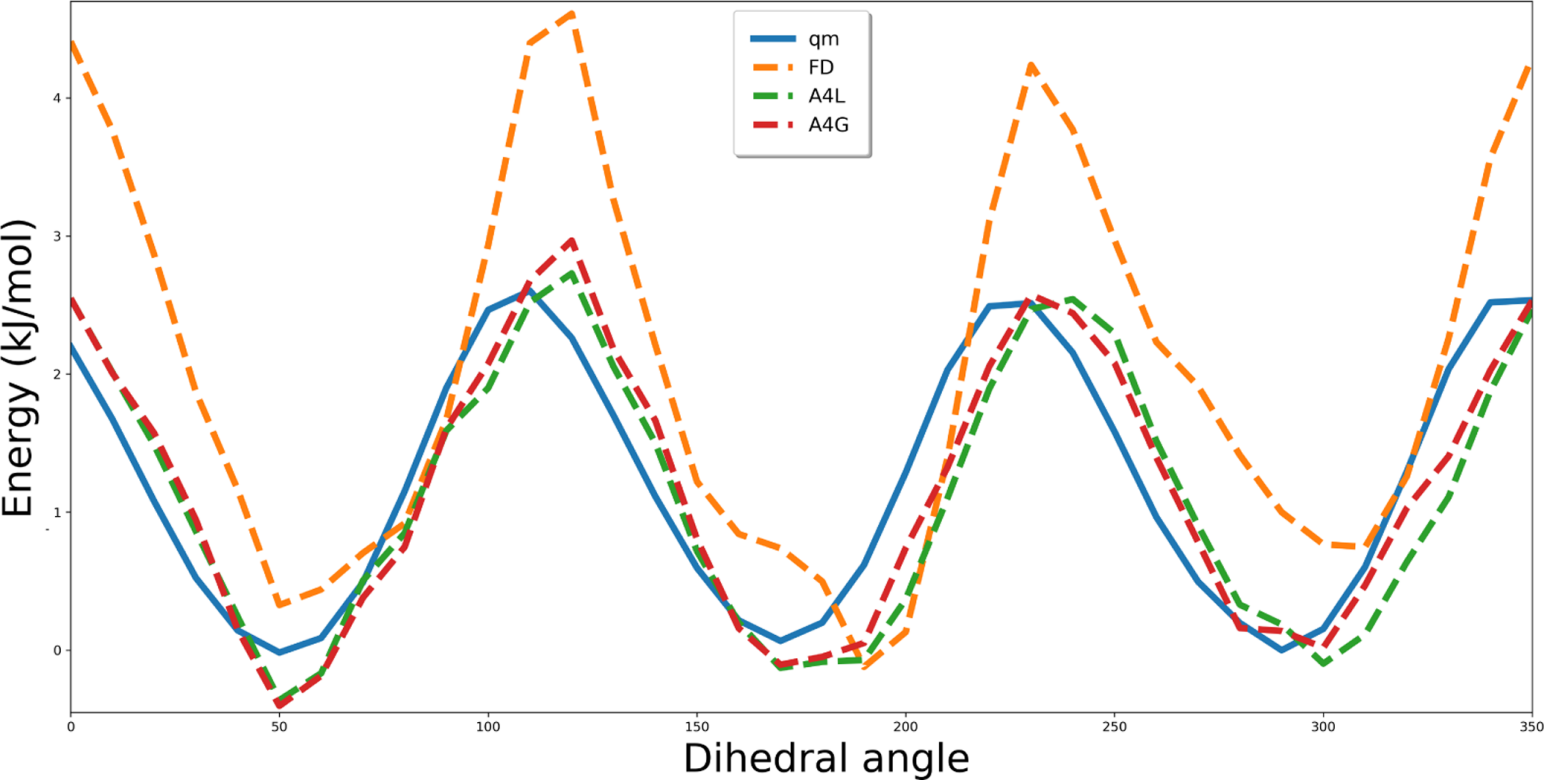
Energy profiles for the HC-CT-OS-P dihedral angle. FD refers to the results of fitting of Fourier coefficients based on the Monte Carlo simulations using the *fit_dihedral.py* program.^14^ A4L (set 3 in Table 2) and A4G (set 4 in Table 2) were obtained using the four-parameter algebraic method (local and global, respectively). FD, A4L, and A4G energy profiles derived from MMs calculations performed with employment of particular sets of parameters.

**Figure 4:**
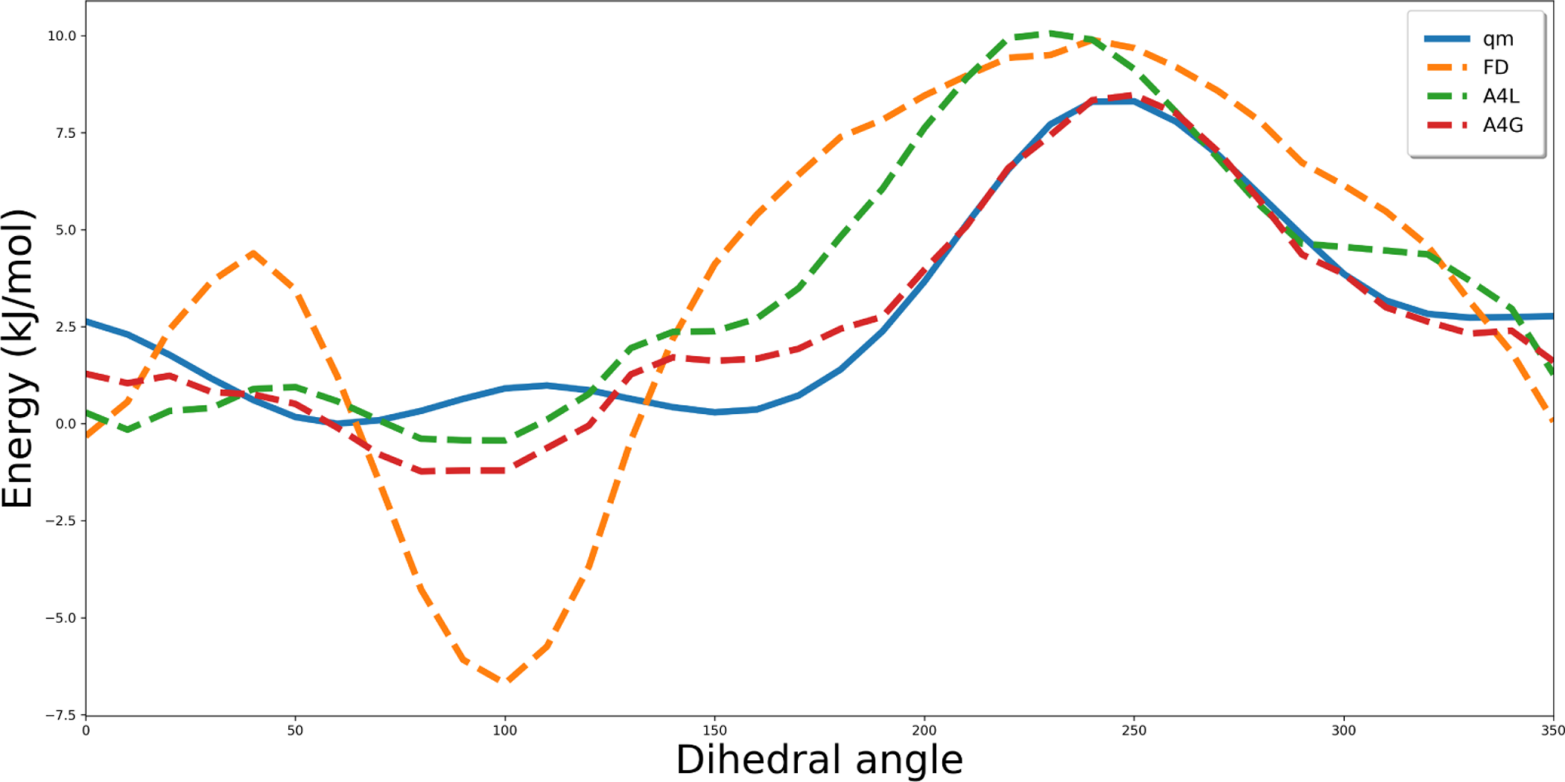
Energy profiles for the HO-OH-P-O2 dihedral angle. FD refers to the results of fitting of Fourier coefficients based on the Monte Carlo simulations using the *fit_dihedral.py* program.^14^ A4L (set 3 in Table 2) and A4G (set 4 in Table 2) were obtained using the four-parameter algebraic method (local and global, respectively). FD, A4L, and A4G energy profiles derived from MMs calculations performed with employment of particular sets of parameters.

Figures 3 and 4 present sample energy profiles for HC-CT-OS-P and HO-OH-P-O2 dihedral types computed with applying van der Waals parameters from original OPLS-AA and Q0 partial charges (type set 1 in Table 2). The comparison of energy profiles for these two dihedral angles obtained for torsional parameters derived from algebraic and MC methods (*fit_dihedral.py* program, labeled as FD) with respect to the QM profiles reveals that the best quality fitting (with the lowest rmsd values) was achieved via an algebraic approach using global minimization procedure. Similar conclusions have been drawn from the comparison of energy profiles obtained for the remaining dihedral angles, which are gathered in Supporting Information.

**Figure 5:**
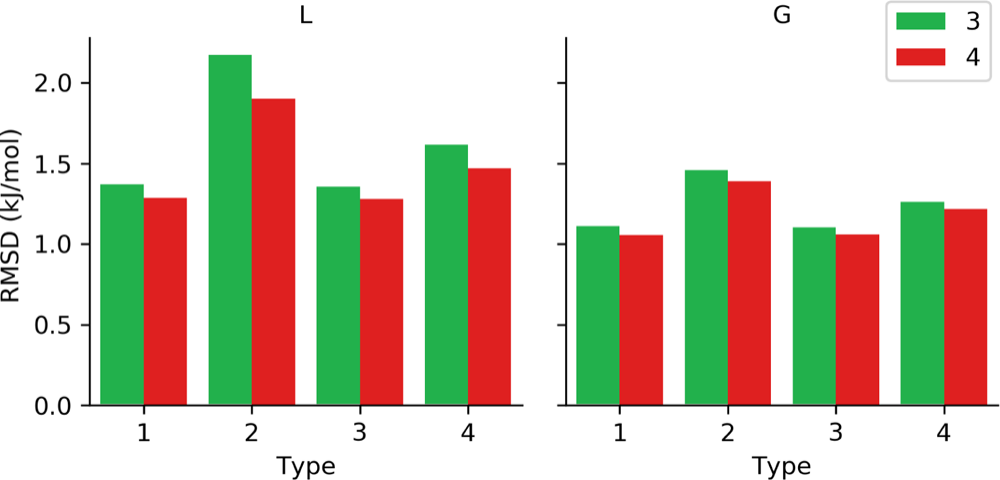
Average rmsd values for profiles obtained with each set type with respect to QM profiles. We observe, as expected, that fitting four Fourier parameters for each dihedral type results in smaller values of rmsd compared to results obtained for three coefficients. Global minimization (G) provides coefficients of better quality than the local one (L).

**Figure 6:**
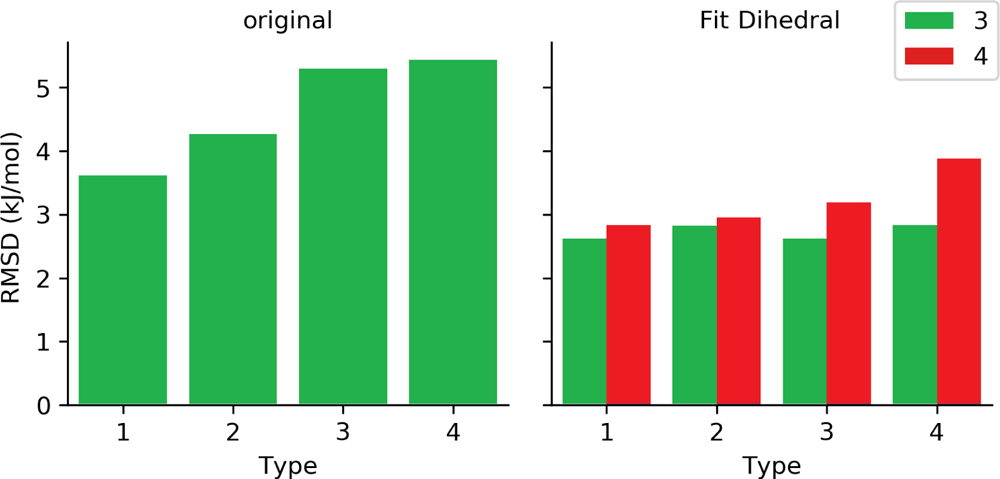
Average rmsd values for MM profiles obtained for each set type containing original coefficients or those obtained using MC simulations (FD) respect to QM profiles.

In order to assess the quality of dihedral parameters assigned with our algebraic method, we calculated average rmsd values for MM energy profiles obtained with each set of considered parameters for all dihedral angles with respect to the corresponding energy profiles computed with QM methods. In Figure 5, we present average rmsd values for algebraically fitted torsional parameters, while in Figure 6, we present average rmsd values for parameters estimated by MC simulations (*fit_dihedral.py* program) and for original OPLS-AA coefficients of torsional potential.

Comparing average rmsd values of MM profiles for different parameter sets with respect to QM results, both approaches, that is, algebraic and MC simulations, significantly improve the description of torsional potential for DMPH with respect to the OPLS-AA original set of parameters. For the algebraic method, the lowest rmsd values were obtained, remaining in the range from 1.06 to 2.17 kJ/mol, while torsional parameters assigned with the MC method causes worse reproduction of energy profiles for rotation of dihedral angles leading to rmsd of 2.62–3.88 kJ/mol. Moreover, results obtained for both approaches revealed that Q0 partial charges assigned based on the QM calculations and RESP procedure, used in set types 1 and 3 (Table 2), allowed us to achieve better reproduction of torsional profiles than Qopls and M.

Selected sets of assigned parameters (Table 2) were also tested in MD simulations, in order to check their ability to reproduce condensed phase properties of the DMPH molecule such as density and enthalpy of vaporization. The results are collected in Table 3.

**Table 3:** Condensed Phase Properties and Related Relative Errors Computed for Each Set (Comparing to Reference Values)^a^

set name	density [kg/m3]	relative error [%]	*d*H_vap_ [kJ/mol]	relative error [%]
set 1	1419.12	7.27	95.27	117.91
set 2	-	-	-	-
set 3	1414.73	6.93	96.69	121.16
set 4	1368.43	3.43	79.91	82.78
set 5	1434.66	8.44	139.54	219.17
set 6	1448.11	9.46	134.14	206.82
set 7	-	-	-	-
set 8	-	-	-	-
set 9	1362.71	3.00	86.86	98.67
set 10	-	-	-	-
set 11	1357.2	2.59	82.84	89.48
set 12	1303.48	1.48	67.03	53.32
set 13	-	-	-	-
set 14	-	-	-	-
set 15	1393.84	5.35	105.52	141.35
set 16	-	-	-	-
reference	1323	-	43.72	-

a The reference density of DMPH derived from www.chemicalbook.com, while reference enthalpy of vaporization was predicted by ACD/Labs.^19^

The best reproduction of liquid density and enthalpy of vaporization was obtained for the same set of parameters, labeled as set 12, containing Q0 partial charges, van der Waals parameters proposed by Murzyn et al.,^19^ and torsional parameters fitted by algebraic methods without constraints imposed on a range of Fourier coefficient values. Condensed phase properties gathered in Table 3 confirm the observation made from the evaluation of reproduction of QM profiles that parameter sets employing Q0 partial charges better reproduce physiochemical properties of the DMPH molecule than considered Qopls and M sets. Two parameters sets, that is, set 4 and set 12 (Table 2) which revealed the best reproduction of QM profiles and condensed phase properties, were additionally tested in reproduction of 540 conformers obtained from QM geometry optimizations. Geometry optimization with strong restraints imposed on a particular dihedral angle revealed that all QM geometries are stable in provided FF, and only negligible changes are observed within coordinates giving the all-atom rmsd in the range of 0.000–0.008 Å.

## 4 Conclusions

An algebraic approach was used to assign Fourier coefficients to four sets of partial charges along with van der Waals parameters in order to improve the description of interatomic and intermolecular interactions in DMPH parameter set provided by OPLS-AA FF. Additionally, we also tested new partial charges, derived from QM calculations and RESP procedure. We compared the results of our algebraic method of torsional parameters assignment with the results derived from Monte Carlo simulations implemented in the *fit_dihedral.py* program.^14^ The present study showed that the algebraic approach we proposed gives better quality results in fitting torsional parameters to reference profiles than MC methods (Figures 3 and 4). Furthermore, one can observe that applying Q0 charges leads to the lowest values of rmsd computed for torsional energy profiles with respect to QM reference (see Figure 5, set type 1 and 3), which has its reflection in the best reproduction of condensed phase properties such as density and enthalpy of vaporization. Importantly, the type of minimization (“global” or “local”) and the number of parameters per dihedral type (4 or 3) have a significant impact on the result. Despite the fact that global minimization with four parameters gives the smallest rmsd with respect to QM reference, they may not always be useful during MD simulations applying the Gromacs package. Large values of torsional parameters cause huge instability of energy during MD simulations and thus errors occur. In many cases, it was not possible to perform NVT or NPT ensembles. According to Table 3, the best reproduction of experimentally known properties of condensed phase of DMPH was obtained for set type 3 (M van der Waals parameters and Q0 charges). Condensed phase properties are reproduced with the lowest relative error with respect to the experimental reference in set 12. The density value is 1303.5 kg/m^3^ (reference-experimental value: 1323 kg/m^3^), and enthalpy of vaporization is 67.03 kJ/mol (reference-experimental value: 43.72 kJ/mol). For original OPLS-AA parameters, they equal 1353 kg/m^3^ and 128.28 kJ/mol, respectively.^19^ Significant improvement of reproduction of enthalpy of vaporization was achieved. The error decreased from 195% for original OPLS-AA and 113% for its modification reported by Murzyn et al.^19^ to 53% obtained in the present study for set 12 (Table 3), respectively. However, it still remains sizable, which might be a consequence of limitations of FF methods, such as constant charges for all conformers observed during the MD simulations, inability of spontaneous protonation, and deprotonation of model molecules in the condensed phase and limited number of provided atom types.

The obtained results showed that the presented algebraic procedure for torsional coefficient fitting as an optimal solution gives better reproduction of QM energy profiles than the Monte Carlo-based method. The latter has many applications, however, as a non-deterministic approach it is time-consuming, demands many outer parameters, and the quality of a solution is difficult to verify. The presented algebraic approach is computationally inexpensive and provides exact solution without additional parameters. Therefore, the limitations of this method are associated with the quality of provided data such as MM energies that depend on FFs parameters other than torsional coefficients, QM energies, and number of conformers that provide sufficient exploration of potential energy surface for the considered molecule. Finding the optimal problem’s solution allows us to observe that the quality of fitting significantly depends on the assigned partial charges. It was proved that the refinement of torsional potential parametrization results in the improvement of intermolecular interaction description, which strongly influences on proper reproduction of condensed phase properties.

## Supporting Information Available

Github repository containing K_fit.py program and its description (https://github.com/mysarapa/K_fit); all sets of parameters considered in this study; and figures presenting selected energy profiles for each dihedral angle obtained with tested sets of parameters (PDF)

## Abbreviations

QM: quantum mechanical methods
MD: molecular dynamics
MM: molecular mechanics
DMPH: dimethyl phosphate
OPLS-AA: optimized potentials for liquid simulations force field, all atom
FF: force field
RB: Ryckaert-Bellemans function
rmsd: root-mean-square deviation
FD: fit_dihedral.py program
RESP: restrained electrostatic potential
NVT: canonical ensemble
NPT: isothermal–isobaric ensemble
