# Supplementary material for: Optimal Solution to the Torsional Coefficient Fitting Problem in Force Field Parametrization
Source: J Phys Chem A. 2021 Mar 24;125(12):2673–81. doi: 10.1021/acs.jpca.0c10845 (PMC8041298; doi:10.1021/acs.jpca.0c10845)
Supplement: Supplementary file 1 — jp0c10845_si_001.pdf [file jp0c10845_si_001.pdf]

Supporting Information for Publication:
“Optimal Solution to Torsional Coefficients Fitting Problem in Force Fields Parametrization”

Adrian Kania, Krzysztof Sarapata, Michał Gucwa, Anna Wójcik-Augustyn*

Department of Computational Biophysics and Bioinformatics, Faculty of Biochemistry, Biophysics and Biotechnology, Jagiellonian University ul. Gronostajowa 7, 30-387 Cracow, Poland

1. The github repository containing *K_fit.py* program and its description:
https://github.com/mysarapa/K_fit

2. Sample of energy profiles for all dihedral angles comparing torsional coefficients derived from *K_fit.py* (algebraic approach) and *fit_dihedral.py* (Monte Carlo method) programs.

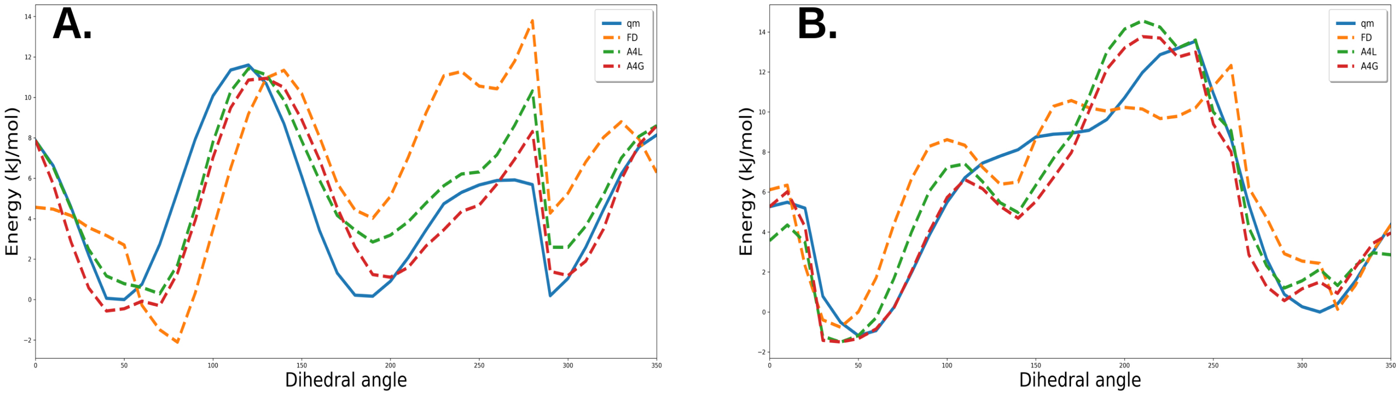

Figure S1: Energy profiles for two dihedral angles in dimethyl phosphoric acid of CT-OS-P-OS type. FD refers to the results of fitting of Fourier coefficients based on the Monte Carlo simulations using fitdihedral.py program (Guvench, 2008). A4L (set 3 in Table 1) and A4G (set 4 in Table 1) were obtained using four-parameter algebraic method (local and global, respectively).

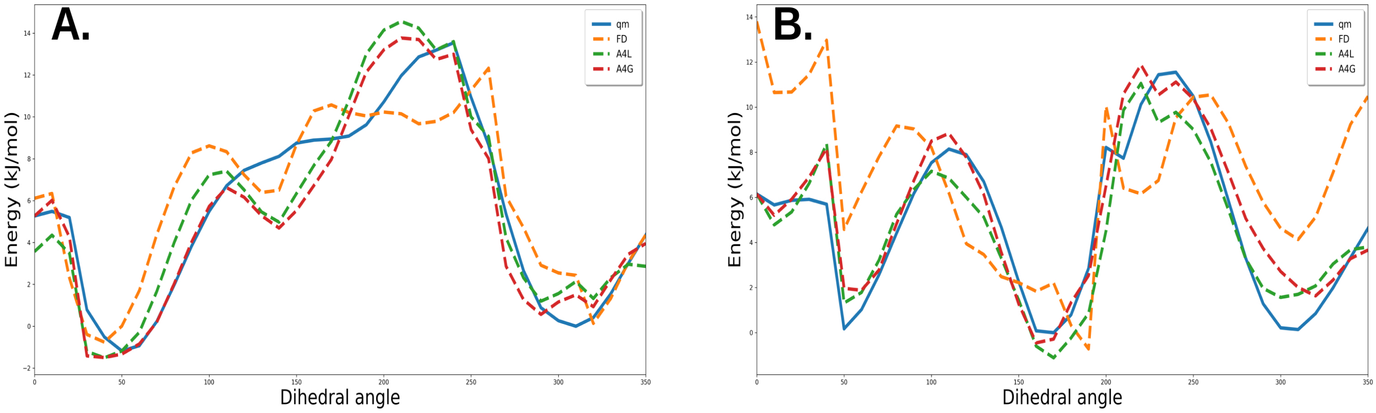

Figure S2: Energy profiles for two dihedral angles in dimethyl phosphoric acid of CT-OS-P-O2 type. FD refers to the results of fitting of Fourier coefficients based on the Monte Carlo simulations using fitdihedral.py program (Guvench, 2008). A4L (set 3 in Table 1) and A4G (set 4 in Table 1) were obtained using four-parameter algebraic method (local and global, respectively).

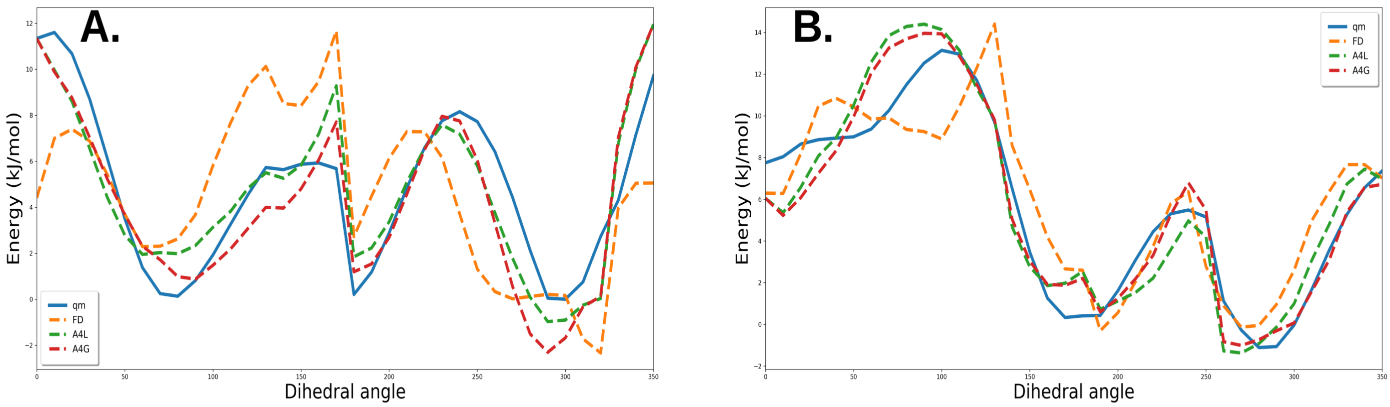

Figure S3: Energy profiles for two dihedral angles in dimethyl phosphoric acid of CT-OS-P-OH type. FD refers to the results of fitting of Fourier coefficients based on the Monte Carlo simulations using fitdihedral.py program (Guvench, 2008). A4L (set 3 in Table 1) and A4G (set 4 in Table 1) were obtained using four-parameter algebraic method (local and global, respectively).

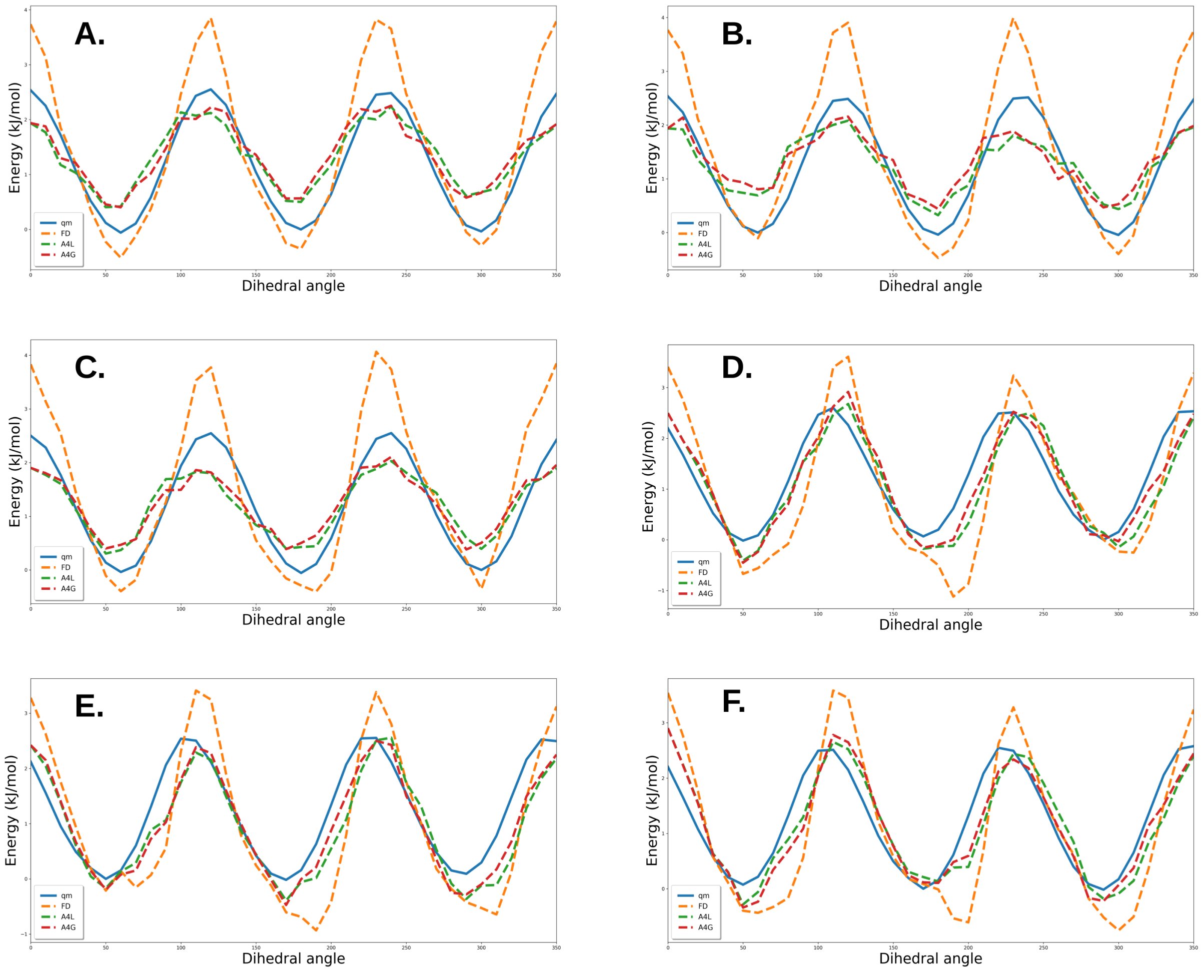

Figure S4: Energy profiles for six dihedral angles in dimethyl phosphoric acid of HC-CT-OS-P type. FD refers to the results of fitting of Fourier coefficients based on the Monte Carlo simulations using fitdihedral.py program (Guvench, 2008). A4L (set 3 in Table 1) and A4G (set 4 in Table 1) were obtained using four-parameter algebraic method (local and global, respectively).

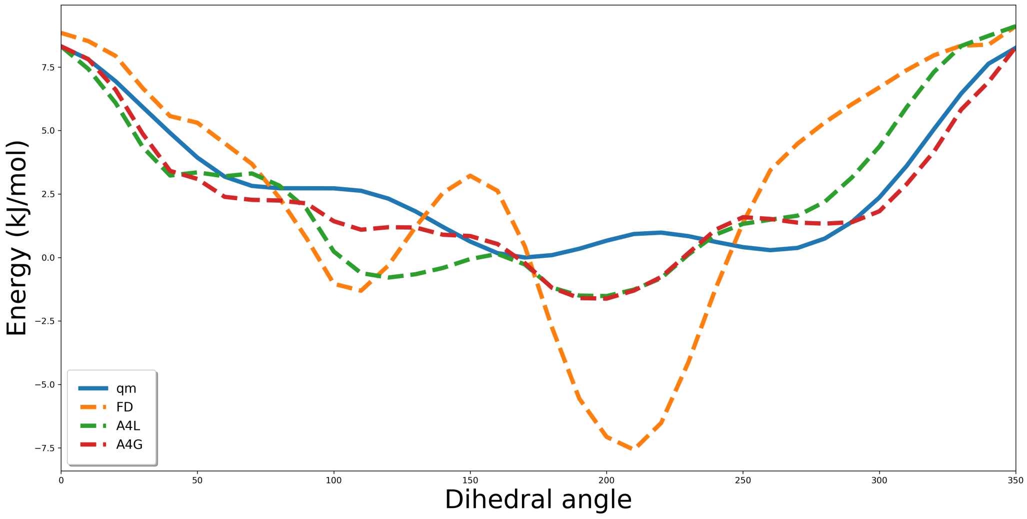

Figure S5: Energy profiles for dihedral angle in dimethyl phosphoric acid of HO-OH-P-O2 type. FD refers to the results of fitting of Fourier coefficients based on the Monte Carlo simulations using fitdihedral.py program (Guvench, 2008). A4L (set 3 in Table 1) and A4G (set 4 in Table 1) were obtained using four-parameter algebraic method (local and global, respectively).

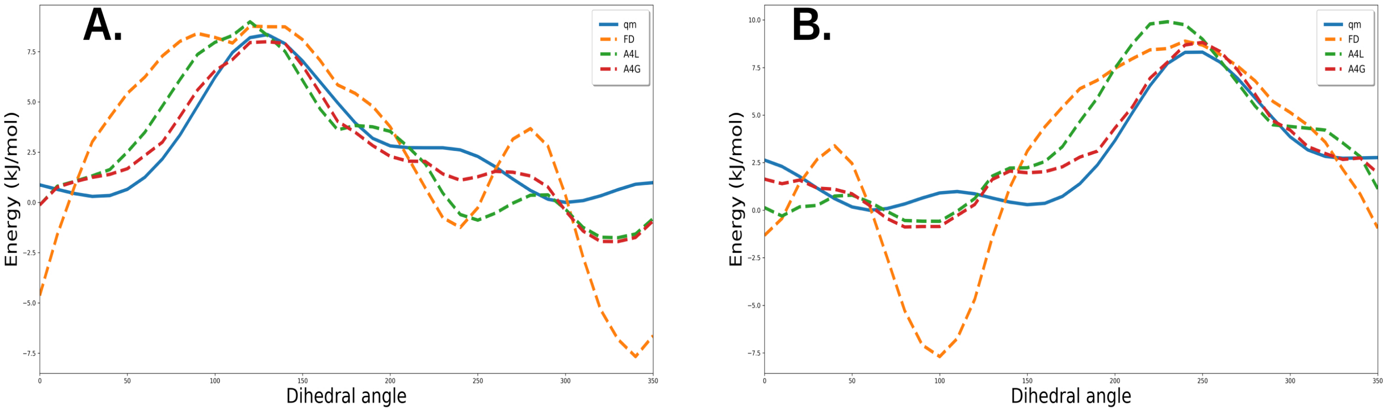

Figure S6: Energy profiles for two dihedral angles in dimethyl phosphoric acid of HO-OH-P-OS type. FD refers to the results of fitting of Fourier coefficients based on the Monte Carlo simulations using fitdihedral.py program (Guvench, 2008). A4L (set 3 in Table 1) and A4G (set 4 in Table 1) were obtained using four-parameter algebraic method (local and global, respectively).

Table 1. Considered sets of parameters.

Set name	Set type	vdW parameters	Charges values	Minimization type	Number of parameters
Set1	1	ORG	Q0	L	3
Set2	1	ORG	Q0	G	3
Set3	1	ORG	Q0	L	4
Set4	1	ORG	Q0	G	4
Set5	2	ORG	Qopls	L	4
Set6	2	ORG	Qopls	L	3
Set7	2	ORG	Qopls	G	4
Set8	2	ORG	Qopls	G	3
Set9	3	M	Q0	L	3
Set10	3	M	Q0	G	3
Set11	3	M	Q0	L	4
Set12	3	M	Q0	G	4
Set13	4	M	M	L	3
Set14	4	M	M	G	3
Set15	4	M	M	L	4
Set16	4	M	M	G	4

Summarizing, 16 sets of parameters were tested. There were 4 types of set differing in van der Waals parameters and partial charges. We performed both local (L) and global (G) minimization searching for 3 and 4 Fourier coefficients for each dihedral type.

3. Sets of partial charges and van der Waals parameters used in the study:

Q0

nonbonded_atom type	bonded_atom_type	charges
opls_135	CT	-0.045
opls_140	HC	0.096
opls_140	HC	0.096
opls_140	HC	0.096
opls_395	OS	-0.331
opls_393	P	0.945
opls_394	O2	-0.550
opls_268	OH	-0.652
opls_270	HO	0.433
opls_395	OS	-0.331
opls_135	CT	-0.045
opls_140	HC	0.096
opls_140	HC	0.096
opls_140	HC	0.096

Qopls

nonbonded_atom type	bonded_atom_type	charges
opls_135	CT	0.190
opls_140	HC	0.060
opls_140	HC	0.060
opls_140	HC	0.060
opls_395	OS	-0.470
opls_393	P	1.270
opls_394	O2	-0.790
opls_268	OH	-0.700
opls_270	HO	0.420
opls_395	OS	-0.470
opls_135	CT	0.190
opls_140	HC	0.060
opls_140	HC	0.060
opls_140	HC	0.060

M

nonbonded_atom type	bonded_atom_type	charges
opls_443	CT	0.430
opls_444	HC	-0.030
opls_444	HC	-0.030
opls_444	HC	-0.030
opls_442	OS	-0.470
opls_440	P	1.270
opls_441	O2	-0.790
opls_268	OH	-0.640
opls_270	HO	0.420
opls_442	OS	-0.470
opls_443	CT	0.430
opls_444	HC	-0.030
opls_444	HC	-0.030
opls_444	HC	-0.030

```
vdW parameters - M
                          sigma         epsilon
 opls_440   P              3.74000e-01  0.58576
 opls_441   O2             3.15000e-01  0.71128
 opls_442   OS             2.90000e-01  0.50208
 opls_268   OH             3.10000e-01  0.58576
```

4. Sets of coefficients of Ryckeart Bellmans potential applied in the study:

```
RB for DIHEDRALS
-------------------------------------------------------------------------
assigned with K_fit.py
```

SET TYPE 1

```
 SET1 (ORIG, Q0, L-3)------------RMSD= 1.3700 kJ/mol
CT    OS     P     OS     3     7.56  -36.00  -40.00   28.44   0.00  0.00
CT    OS     P     OH     3     7.92  -48.68  -37.64   40.76   0.00  0.00
CT    OS     P     O2     3    38.59   35.77  -25.82  -74.36   0.00  0.00
HC    CT    OS     P      3    -7.21    7.97  -19.30   -0.76   0.00  0.00
HO    OH     P     O2     3   -18.32    3.91  -29.62   14.40   0.00  0.00
HO    OH     P     OS     3   -16.96   29.12  -39.66  -12.16   0.00  0.00

 SET2 (ORIG, Q0, G-3)------------RMSD= 1.1110 kJ/mol
CT    OS     P     OS     3    30.47  -20.07  -35.94  -10.40   0.00  0.00
CT    OS     P     OH     3    32.06  -32.22  -31.96    0.16   0.00  0.00
CT    OS     P     O2     3    35.59  -33.47  -23.24   -2.12   0.00  0.00
HC    CT    OS     P      3     6.23   -5.79   -0.56   -0.44   0.00  0.00
HO    OH     P     O2     3   -90.16  103.84  -39.38  -13.68   0.00  0.00
HO    OH     P     OS     3   -89.18   86.90  -55.58    2.28   0.00  0.00

 SET3 (ORIG, Q0, L-4)------------RMSD= 1.2850 kJ/mol
CT    OS     P     OS     3     4.64  -35.98  -27.92   28.32 -12.08  0.00
CT    OS     P     OH     3     6.92  -45.52  -26.82   36.00 -10.40  0.00
CT    OS     P     O2     3    31.77   32.35   -2.78  -69.80 -22.72  0.00
HC    CT    OS     P      3   -13.97   20.75  -27.00   -1.00  23.12  0.00
HO    OH     P     O2     3    -4.14   -8.50 -103.68   30.96  73.28  0.00
HO    OH     P     OS     3    -2.91   36.29  -86.64  -21.72  46.64  0.00

 SET4 (ORIG, Q0, G-4)------------RMSD= 1.0570 kJ/mol
CT    OS     P     OS     3    12.23  -24.00  -23.70    5.28 -27.68  0.00
CT    OS     P     OH     3    12.29  -33.03  -13.42   12.36 -33.52  0.00
CT    OS     P     O2     3    19.43   -5.05   20.86  -27.48 -52.40  0.00
HC    CT    OS     P      3    22.18  -18.38  -14.32   -0.56  12.96  0.00
HO    OH     P     O2     3   -69.90  109.12  -79.86  -28.20  44.08  0.00
HO    OH     P     OS     3   -78.86   77.34  -80.88    9.08  30.24  0.00

-------------------------------------------------------------------------
```

SET TYPE 2

```
 SET5 (ORIG, Qopls, L-4)------------RMSD= 1.9000 kJ/mol
CT    OS     P     OS     3    -4.82  -36.84  -12.72   34.84 -27.28  0.00
CT    OS     P     OH     3    -2.57  -46.67  -17.30   44.08 -20.64  0.00
CT    OS     P     O2     3    27.60   40.78   20.00  -80.00 -46.48  0.00
HC    CT    OS     P      3    13.59  -12.13    6.18    0.44   7.60  0.00
HO    OH     P     O2     3    14.70  -30.64 -183.06   53.80 151.44  0.00
HO    OH     P     OS     3    12.48   45.86 -134.28  -34.48  95.44  0.00
```

SET6 (ORIG, Qopls, L-3)------------RMSD= 2.1710 kJ/mol

CT	OS	P	OS	3	2.47	-33.75	-17.36	31.28	0.00	0.00
CT	OS	P	OH	3	1.78	-50.38	-16.10	48.60	0.00	0.00
CT	OS	P	O2	3	40.00	40.00	-13.80	-80.00	0.00	0.00
HC	CT	OS	P	3	19.97	-20.09	-12.74	0.12	0.00	0.00
HO	OH	P	O2	3	-22.34	-25.54	-31.72	47.88	0.00	0.00
HO	OH	P	OS	3	-12.62	42.14	-40.00	-29.52	0.00	0.00

SET7 (ORIG, Qopls, G-4)------------RMSD= 1.3890 kJ/mol

CT	OS	P	OS	3	25.73	-48.93	-13.74	13.56	-38.56	0.00
CT	OS	P	OH	3	25.29	-59.99	-2.14	23.40	-45.20	0.00
CT	OS	P	O2	3	45.35	-17.13	42.62	-46.60	-73.52	0.00
HC	CT	OS	P	3	55.44	-53.04	1.80	0.24	10.56	0.00
HO	OH	P	O2	3	-111.91	161.21	-89.48	-36.00	53.20	0.00
HO	OH	P	OS	3	-123.03	118.71	-88.64	13.40	36.32	0.00

SET8 (ORIG, Qopls, G-3)------------RMSD= 1.4590 kJ/mol

CT	OS	P	OS	3	53.30	-41.18	-33.72	-12.12	0.00	0.00
CT	OS	P	OH	3	54.15	-57.03	-29.34	2.88	0.00	0.00
CT	OS	P	O2	3	67.47	-62.87	-21.22	-4.60	0.00	0.00
HC	CT	OS	P	3	37.38	-37.62	6.88	0.24	0.00	0.00
HO	OH	P	O2	3	-140.84	154.96	-38.38	-14.12	0.00	0.00
HO	OH	P	OS	3	-137.85	134.81	-55.08	3.04	0.00	0.00

SET TYPE 3

SET9 (M, Q0, L-3)----------------RMSD= 1.3550 kJ/mol

CT	OS	P	OS	3	7.99	-37.91	-40.00	29.92	0.00	0.00
CT	OS	P	OH	3	7.73	-49.93	-38.70	42.20	0.00	0.00
CT	OS	P	O2	3	39.29	37.87	-26.74	-77.16	0.00	0.00
HC	CT	OS	P	3	11.44	-11.72	16.96	0.28	0.00	0.00
HO	OH	P	O2	3	-18.63	3.79	-29.28	14.84	0.00	0.00
HO	OH	P	OS	3	-16.90	29.30	-39.86	-12.40	0.00	0.00

SET10 (M, Q0, G-3)----------------RMSD= 1.1030 kJ/mol

CT	OS	P	OS	3	31.55	-20.75	-35.54	-10.80	0.00	0.00
CT	OS	P	OH	3	32.49	-32.13	-32.70	-0.36	0.00	0.00
CT	OS	P	O2	3	35.63	-34.43	-23.98	-1.20	0.00	0.00
HC	CT	OS	P	3	39.73	-39.21	3.34	-0.16	0.00	0.00
HO	OH	P	O2	3	-87.20	101.40	-39.12	-14.20	0.00	0.00
HO	OH	P	OS	3	-86.56	84.04	-55.72	2.52	0.00	0.00

SET11 (M, Q0, L-4)----------------RMSD= 1.2780 kJ/mol

CT	OS	P	OS	3	5.43	-36.05	-27.12	27.40	-12.88	0.00
CT	OS	P	OH	3	7.19	-45.07	-27.78	35.24	-10.56	0.00
CT	OS	P	O2	3	31.08	31.16	-3.00	-68.12	-23.52	0.00
HC	CT	OS	P	3	0.48	3.82	21.52	0.32	18.48	0.00
HO	OH	P	O2	3	-4.90	-7.56	-98.78	29.68	68.88	0.00
HO	OH	P	OS	3	-3.66	35.50	-83.52	-20.92	43.68	0.00

SET12 (M, Q0, G-4)----------------RMSD= 1.0600 kJ/mol

CT	OS	P	OS	3	14.01	-25.01	-23.06	4.36	-26.56	0.00
CT	OS	P	OH	3	13.67	-33.17	-14.78	11.60	-31.6	0.00
CT	OS	P	O2	3	20.37	-6.99	18.28	-25.84	-49.84	0.00
HC	CT	OS	P	3	54.23	-50.89	-8.94	-0.28	12.24	0.00
HO	OH	P	O2	3	-68.67	106.33	-74.38	-28.12	38.16	0.00
HO	OH	P	OS	3	-77.28	74.66	-77.92	9.16	26.16	0.00

<u>SET TYPE 4</u>

SET13 (M, M, L-3)----------------RMSD= 1.6160 kJ/mol

CT	OS	P	OS	3	5.63	-37.79	-35.80	32.16	0.00	0.00
CT	OS	P	OH	3	2.02	-45.90	-35.60	43.88	0.00	0.00
CT	OS	P	O2	3	39.93	39.79	-25.58	-79.72	0.00	0.00
HC	CT	OS	P	3	19.95	-20.15	1.92	0.20	0.00	0.00
HO	OH	P	O2	3	-16.14	-2.86	-29.38	19.00	0.00	0.00
HO	OH	P	OS	3	-16.28	31.16	-40.00	-14.88	0.00	0.00

SET14 (M, M, G-3)----------------RMSD= 1.2620 kJ/mol

CT	OS	P	OS	3	41.85	-25.97	-34.14	-15.88	0.00	0.00
CT	OS	P	OH	3	39.50	-33.82	-31.86	-5.68	0.00	0.00
CT	OS	P	O2	3	46.18	-53.66	-23.64	7.48	0.00	0.00
HC	CT	OS	P	3	40.94	-40.62	-7.62	-0.32	0.00	0.00
HO	OH	P	O2	3	-102.88	112.68	-31.12	-9.80	0.00	0.00
HO	OH	P	OS	3	-101.91	101.31	-45.42	0.60	0.00	0.00

SET15 (M, M, L-4)----------------RMSD= 1.4680 kJ/mol

CT	OS	P	OS	3	-0.74	-39.92	-16.72	34.84	-23.28	0.00
CT	OS	P	OH	3	-1.47	-43.97	-19.74	40.56	-19.52	0.00
CT	OS	P	O2	3	29.40	39.40	14.22	-79.20	-41.6	0.00
HC	CT	OS	P	3	4.14	0.50	19.64	0.44	20.32	0.00
HO	OH	P	O2	3	2.43	-12.31	-115.94	31.36	85.92	0.00
HO	OH	P	OS	3	-0.85	36.69	-94.24	-22.28	54.24	0.00

SET16 (M, M, G-4)----------------RMSD= 1.2170 kJ/mol

CT	OS	P	OS	3	19.90	-31.33	-18.44	3.72	-30.88	0.00
CT	OS	P	OH	3	16.56	-35.42	-11.56	10.08	-35.12	0.00
CT	OS	P	O2	3	28.16	-17.94	25.80	-24.60	-57.52	0.00
HC	CT	OS	P	3	56.96	-52.64	-20.72	-0.48	15.36	0.00
HO	OH	P	O2	3	-79.10	116.18	-72.16	-26.20	43.52	0.00
HO	OH	P	OS	3	-89.22	88.28	-71.60	8.36	29.68	0.00

The best RB parameters obtained from *fit_dihedral.py* program: for type set 1 (original vdW and Q0 charges) RMSD=2.623kJ/mol

CT	OS	P	OS	3	-1.31	-1.67	-30.52	0.68	-9.20	0.00
CT	OS	P	OH	3	2.75	1.93	-30.36	-6.60	-7.68	0.00
CT	OS	P	O2	3	3.72	-4.02	-27.88	-0.44	-2.96	0.00
HC	CT	OS	P	3	51.00	-15.52	-120.16	-2.68	131.20	0.00
HO	OH	P	O2	3	54.19	27.23	-112.46	-55.24	104.72	0.00
HO	OH	P	OS	3	17.88	-27.46	-72.46	27.48	71.60	0.00
